# Acesso Radial e Fondaparinux: Uma Interação Sinérgica

**DOI:** 10.36660/abc.20250556

**Published:** 2025-11-13

**Authors:** Pedro Beraldo de Andrade, Leonardo Maróstica Alves Silva

**Affiliations:** 1 Santa Casa de Marília São Paulo SP Brasil Santa Casa de Marília, São Paulo, SP – Brasil

**Keywords:** Infarto do Miocárdio sem Supradesnivelamento do Segmento ST, Fondaparinux, Artéria Radial

A consolidação do tratamento antitrombótico baseado na terapia antiplaquetária dupla com ácido acetilsalicílico e inibidores do receptor P2Y_12_,^1^ a adoção rotineira da estratificação de risco invasiva,^2^ o refinamento técnico da intervenção coronária percutânea (ICP), com maior utilização de imagem intravascular como ferramenta adjunta para guiar os procedimentos,^3^ assim como a evolução tecnológica dos stents coronários,^4^ culminaram com um declínio temporal nas duas últimas décadas na ocorrência de complicações isquêmicas de pacientes admitidos com síndrome coronariana aguda sem supradesnivelamento do segmento ST (SCASSST).^5^ Nesse contexto, o risco de sangramento deixou de ser tolerado como uma intercorrência inerente ao tratamento anti-isquêmico, passando a desempenhar um papel central na busca pelo equilíbrio do binômio eficácia e segurança na condução clínica desse perfil de pacientes. O protagonismo do risco hemorrágico foi alcançado a partir de publicações demonstrando que sangramentos graves, fossem aqueles relacionados à via de acesso arterial utilizada na ICP, ou sangramentos do sistema nervoso central, trato digestivo, aparelho gênito-urinário, associavam-se a um pior prognóstico, assemelhando-se ou superando em morbimortalidade a recorrência de eventos isquêmicos.^6,7^

Em 2010 emergiu então o conceito de *Bleeding Avoidance Strategies*,^8^ consistindo em um conjunto de medidas associadas a uma prática clínica de qualidade, custo-efetiva, cujas métricas evidenciam sua capacidade de mitigar as taxas de sangramento grave em pacientes submetidos à estratificação de risco invasiva (Tabela 1).^9,10^

**Tabela 1:** Linhas de cuidado (Bleeding Avoidance Strategies)

**Manuseio criterioso da farmacoterapia**	Preferência por fondaparinux ou bivaluridina (esta não disponível no país) com terapia anticoagulante. Evitar o uso concomitante de enoxaparina e heparina não-fracionada. Uso judicioso de inibidores do receptor de glicoproteína IIbIIIa (habitualmente reservado à sala de Hemodinâmica). Prescrição racional da terapia antiplaquetária dupla (evitar o pré-tratamento quando há previsão de estratificação nas primeiras 24 horas da admissão; estratégias de descalonamento em pacientes de alto risco de sangramento).
**Cuidados relacionados à via de acesso arterial**	Predileção pelo acesso radial. Uso de introdutores de menor calibre quando pertinente. Remoção precoce do introdutor após o término do procedimento. Quando da opção pelo acesso femoral, emprego da técnica de punção ótima (guiada por ultrassom; guiada por fluoroscopia, na zona de segurança representada pelo 1/3 médio da cabeça do fêmur; micro-punção; uso de dispositivos de oclusão vascular).

Nessa edição, Ritt et al. ilustram o sinergismo da combinação via de acesso radial e terapia anticoagulante com fondaparinux em uma coorte retrospectiva de 956 pacientes submetidos à estratificação de risco invasiva na vigência de uma SCASSST.^11^ Avaliando a taxa intra-hospitalar de eventos adversos graves compostos por morte, reinfarto, acidente vascular encefálico e sangramento grave pelo critério OASIS-5, os autores demonstraram que, em detrimento ao uso do acesso femoral, a opção pelo acesso radial promoveu um redução significativa de 54% no desfecho primário, ao passo que a utilização de fondaparinux reduziu em 43% a ocorrência de eventos comparada à prescrição de enoxaparina. Pacientes nos quais a via de acesso radial e a terapia com fondaparinux foram selecionadas (n=366) apresentaram taxa do desfecho primário de 3,3%, cotejada com uma taxa de 14,4% no grupo que utilizou a via de acesso femoral juntamente com enoxaparina (p<0,001).

Os autores merecem congratulações pela publicação. Apesar de o estudo ser uma análise não randômica, retrospectiva, houve o cuidado em buscar individualmente informações acerca da evolução intra-hospitalar dos participantes da pesquisa. Eventuais vieses na seleção dos pacientes quanto à via de acesso arterial, bem como à terapia antitrombótica, não podem ser descartados, e os grupos diferem entre si quanto a características sabidamente preditoras de maior risco isquêmico e hemorrágico, como idade, insuficiência cardíaca, doença arterial periférica, acidente vascular encefálico prévio, função renal, nível de hemoglobina, uso de inibidores do receptor P2Y_12_ e anticoagulantes orais. Mas a plausibilidade biológica de um efeito sinérgico entre via de acesso radial e fondaparinux sustenta a possibilidade de os achados do estudo representarem uma verdade científica. Em nossa experiência pessoal, a utilização de fondaparinux, suplementada com heparina não-fracionada no momento da ICP, em população similar de pacientes admitidos por SCASSST, na qual o acesso radial foi utilizado em 95,3% dos casos, esteve associada a uma taxa de eventos adversos isquêmicos e de eventos hemorrágicos de 3,2%,^12^ similar ao reportado pelos autores.

Ao eleger a via radial como acesso preferencial no atendimento aos pacientes com SCASSST, sabidamente associada a menor risco de sangramento grave quando comparada à via femoral,^13^ bem como a utilização de fondaparinux em substituição à enoxaparina, minimizando o risco de dosagem supraterapêutica (a dose diária profilática para tromboembolismo venoso, de 2,5 mg subcutâneo, é a mesma utilizada no tratamento da SCASSST), e de cruzamento inadvertido entre heparinas (heparina não-fracionada representa o anticoagulante de eleição nos laboratórios de Hemodinâmica nacionais, sendo sua suplementação requerida entre pacientes tratados com fondaparinux),^14^ oferta-se ao paciente duas importantes linhas de prevenção de eventos hemorrágicos e isquêmicos, atuando de forma sinérgica, como demonstrado na presente publicação.
