# Supplementary material for: Acesso Radial e Fondaparinux: Uma Interação Sinérgica
Source: Arq Bras Cardiol. 2025 Nov 13;122(9):e20250556. [Article in Portuguese] doi: 10.36660/abc.20250556 (PMC12671573; doi:10.36660/abc.20250556)
Supplement: Translation

# Translation: Radial Access and Fondaparinux: A Synergistic Interaction

**DOI:** 10.36660/abc.20250556i

**Authors:** Pedro Beraldo de Andrade, Leonardo Maróstica Alves Silva

**Affiliations:** 1 São Paulo SP Brazil Santa Casa de Marília, São Paulo, SP – Brazil

**Keywords:** Non-ST Elevated Myocardial Infarction, Fondaparinux, Radial Artery

The consolidation of antithrombotic treatment based on dual antiplatelet therapy with aspirin and P2Y_12_ receptor inhibitors,^1^ the routine adoption of invasive risk stratification,^2^ the technical refinement of percutaneous coronary intervention (PCI), with greater use of intravascular imaging as an adjunctive tool to guide procedures,^3^ as well as the technological evolution of coronary stents,^4^ culminated in a temporal decline over the last two decades in the occurrence of ischemic complications in patients admitted with non-ST-segment elevation acute coronary syndrome (NSTEMI).^5^ In this context, the risk of bleeding is no longer tolerated as an inherent complication of anti-ischemic treatment. Still, it has come to play a central role in the search for a balance between efficacy and safety in the clinical management of this patient profile. The importance of bleeding risk was achieved through publications demonstrating that major bleeding, whether related to PCI arterial access or bleeding in the central nervous system, digestive tract, or genitourinary system, was associated with a worse prognosis, with morbidity and mortality similar to or exceeding recurrent ischemic events.^6,7^

In 2010, the concept of "Bleeding Avoidance Strategies" emerged,^8^ consisting of a set of measures associated with high-quality, cost-effective clinical practice, whose metrics demonstrate its ability to mitigate major bleeding rates in patients undergoing invasive risk stratification (Table 1)..^9,10^

**Table 1:** Lines of care (Bleeding Avoidance Strategies)

**Careful management of pharmacotherapy**	Preference for fondaparinux or bivalirudin (not available in Brazil) as anticoagulant therapy. Avoid concomitant use of enoxaparin and unfractionated heparin. Judicious use of glycoprotein IIbIIIa receptor inhibitors (usually reserved for the Cath lab). Rational prescription of dual antiplatelet therapy (avoiding pretreatment when stratification is anticipated within the first 24 hours of admission; de-escalation strategies in patients at high risk of bleeding).
**Care related to arterial access**	Preference for radial access. Use of smaller-caliber sheaths when appropriate. Early removal of the sheath after the end of the procedure. When opting for femoral access, use the optimal puncture technique (ultrasound-guided; fluoroscopy-guided in the safety zone represented by the middle 1/3 of the femoral head; micro-puncture; use of vascular closure devices).

In this issue, Ritt et al. illustrate the synergism of the combination of radial access and anticoagulant therapy with fondaparinux in a retrospective cohort of 956 patients undergoing invasive risk stratification in the presence of NSTEMI.^11^ Evaluating the in-hospital rate of major adverse events, composed of death, reinfarction, stroke, and major bleeding, according to the OASIS-5 criteria, the authors demonstrated that, instead of the use of femoral access, the option for radial access promoted a significant reduction of 54% in the primary outcome, while the use of fondaparinux reduced the occurrence of events by 43% compared to the prescription of enoxaparin. Patients in whom the radial access and fondaparinux therapy were selected (n=366) had a primary outcome rate of 3.3%, compared with a rate of 14.4% in the group that used the femoral access combined with enoxaparin (p<0.001). The authors deserve congratulations on this publication. Although the study was a non-randomized, retrospective analysis, care was taken to individually collect information about the in-hospital outcomes of the study participants. Potential biases in patient selection regarding arterial access and antithrombotic therapy cannot be ruled out, and the groups differed in characteristics known to predict increased ischemic and hemorrhagic risk, such as age, heart failure, peripheral arterial disease, prior stroke, renal function, hemoglobin level, use of P2Y_12_ receptor inhibitors, and oral anticoagulants. However, the biological plausibility of a synergistic effect between radial access and fondaparinux supports the possibility that the study findings represent scientific truth. In our personal experience, the use of fondaparinux, supplemented with unfractionated heparin at the time of PCI, in a similar population of patients admitted for NSTEMI, in which radial access was used in 95.3% of cases, was associated with a rate of ischemic adverse events and bleeding events of 3.2%,^12^ like that reported by the authors.

By choosing the radial approach as the preferred access for treating patients with NSTEMI, known to be associated with a lower risk of major bleeding when compared to the femoral approach,^13^ as well as the use of fondaparinux instead of enoxaparin, minimizing the risk of supratherapeutic dosage (the prophylactic daily dose for venous thromboembolism, 2.5 mg subcutaneously, is the same used in the treatment of NSTEMI), and inadvertent crossover between heparins (unfractionated heparin is the anticoagulant of choice in Brazilian Cath labs, and its supplementation is required for patients treated with fondaparinux),^14^ two important fronts for the prevention of hemorrhagic and ischemic events are offered, which act synergistically, as demonstrated in this publication.
